# Self-rated dental health and dental insurance: modification by household income

**DOI:** 10.1186/1477-7525-12-67

**Published:** 2014-05-09

**Authors:** Dana N Teusner, Olga Anikeeva, David S Brennan

**Affiliations:** 1 ARCPOH, School of Dentistry, University of Adelaide, 122 Frome Street, 5005 Adelaide, South Australia; 2 Disaster Research Centre, School of Nursing and Midwifery, Flinders University, 5001 Bedford Park, South Australia

**Keywords:** Self-rated dental health, Oral health, Dental insurance, Income

## Abstract

**Background:**

Previous studies have reported that socioeconomically disadvantaged Australians have poorer self-rated dental health (SRDH), are less likely to be insured for dental services and are less likely to have regular dental visits than their more advantaged counterparts. However, less is known about the associations between dental insurance and SRDH. The aim of this study was to examine the associations between SRDH and dental insurance status and to test if the relationship was modified by household income.

**Methods:**

A random sample of 3,000 adults aged 30–61 years was drawn from the Australian Electoral Roll and mailed a self-complete questionnaire. Analysis included dentate participants. Bivariate associations were assessed between SRDH and insurance stratified by household income group. A multiple variable model adjusting for covariates estimated prevalence ratios (PR) of having good to excellent SRDH and included an interaction term for insurance and household income group.

**Results:**

The response rate was 39.1% (n = 1,093). More than half (53.9%) of the participants were insured and 72.5% had good to excellent SRDH. SRDH was associated with age group, brushing frequency, insurance status and income group. Amongst participants in the $40,000– < $80,000 income group, the insured had a higher proportion reporting good to excellent SRDH (80.8%) than the uninsured (66.5%); however, there was little difference in SRDH by insurance status for those in the $120,000+ income group. After adjusting for covariates, there was a significant interaction (p < 0.05) between having insurance and income; there was an association between insurance and SRDH for adults in the $40,000– < $80,000 income group, but not for adults in higher income groups.

**Conclusions:**

For lower socio-economic groups being insured was associated with better SRDH, but there was no association for those in the highest income group. Insurance coverage may have the potential to improve dental health for low income groups.

## Introduction

Oral health is recognised as an integral component of general health, with poorer oral health reflected in worse general health and quality of life [1]. Single item global self-ratings are frequently used to measure health status [2]. Global self-ratings of health are non-clinical measures that involve individual perceptions about overall health and permit persons to incorporate their own judgement about how to combine the various dimensions of health [3]. An individual’s assessment of health may include current and/or previous disease experience, but may also include other dimensions such as social impacts of health, functional limitations and health behaviours. Global self-ratings have been used to assess both general health [4,5] and also oral health status and have been used to predict mortality and morbidity, screen for high-risk groups and as endpoints for clinical trials [3].

In Australia, the majority of adults are ineligible for public dental care and must pay for dental services, either by making payments in full or by purchasing dental insurance, which provides partial reimbursement. More than half of the population privately purchase insurance cover for dental services as insurance is rarely provided as part of employment contracts. The range of services covered and the level of rebate provided vary by policy, but on average dental polices provide approximately 50% rebate on dental fees [6]. The degree to which insured people deliberately self-select into dental insurance based on their dental health status, risk perception or personal attitudes is not clear. Firstly, cover for dental services is rarely marketed or purchased separately, nearly all dental cover is purchased as part of a combined hospital and general treatment (extras) package of cover; only small proportions (less than 5%) purchase hospital only cover or general treatment (extras) policies not covering dental care [7]. Secondly, there is a system of subsides and surcharges which provide incentives for the purchase of health insurance. For many working individuals and families there is little financial rationality in remaining uninsured; depending on household income there is little difference in household overheads between purchasing health insurance or paying an additional tax levy incurred as a result of opting out of insurance. Despite the subsidies provided for health insurance premiums, a social gradient in health insurance cover is evident; those in the highest socio-economic quintile are more likely to be insured than those in lower quintiles [7].

Consequently socioeconomically disadvantaged adults face substantial financial barriers to accessing dental care. Adult public dental services in Australia are limited, rationed via triaging systems and long waiting periods, [8] which may contribute to the worsening of existing dental problems. Limited public sector access may lead to restricted treatment options available to public patients and to problem-oriented dental visiting [9,10]. Thus, socioeconomically disadvantaged Australians who cannot afford to pay for dental care or dental insurance are less likely to receive preventive or routine care, resulting in poorer oral health outcomes [8,10].

Previous studies show that income is associated with dental visiting and dental health outcomes, with socioeconomically disadvantaged individuals more likely to report a lower frequency of visiting, a higher frequency of visiting for pain relief, a higher number of extractions and missing teeth and poorer self-rated oral health [8-22]. Individuals from low income households were more likely to report that oral health problems impacted negatively on their quality of life [19,23] and that they experienced greater functional and psychosocial impacts than higher income earners [17], even after controlling for levels of oral disease and impairment [16].

Numerous studies have reported associations between dental insurance and lower rates of extractions, higher rates of visiting for a check-up and regular dental visiting [11-14,21,24-26]. Insured patients faced fewer financial barriers to accessing comprehensive dental care and were more likely to accept the treatment prescribed by their dentist [27]. However, fewer studies have directly assessed the associations between dental insurance and self-rated dental health.

The aim of this study was to examine the association between self-rated dental health and dental insurance status across household income groups. We expected dental insurance to be positively associated with self-rated dental health by reducing financial barriers to timely and comprehensive dental care. Similarly, we expected household income to be positively associated with self-rated dental health. In addition, we explored the relationship between dental insurance and self-rated dental health stratified by household income to investigate whether the anticipated associations varied by socioeconomic status.

## Methods

A random sample of 3,000 adults aged 30–60 years living in Australia was drawn from the Electoral Roll. Data were collected by mailed self-complete questionnaires in 2009–2010, with four follow-up mailings to non-respondents. This age group was selected in order to capture working aged adults; some respondents were older than 60 years at the time of completing the questionnaire but were retained in the study. Sample size was determined by using estimates of percentage of persons making a dental visit in the last year (reflecting access to care), percentage of persons receiving extractions (for comprehensiveness of care), and percentage of persons reporting their self-rated dental health (oral health status). Calculations were made based on comparisons of proportions using an alpha level of 0.05 and a beta of 0.80. The largest required sample size was n = 336 per group for comprehensiveness of care, which, allowing for 3 levels of disaggregation, would require a total of 1,008 subjects.

The research was approved by the Human Research Ethics Committee of the University of Adelaide.

### Outcome variables

The outcome variable was self-rated dental health (SRDH). Self-rated dental health was assessed using a single-item global rating. Conceptually global ratings are considered as general health perceptions in the Wilson and Cleary model for health outcomes [28], which links physiological variables, symptoms, functional health, general health perceptions and overall quality of life [29,30]. The index category were those who reported good, very good or excellent dental health, the reference category were those who reported poor or very poor dental health.

### Explanatory variables

The main explanatory variables were dental insurance status and household income. Dental insurance was coded as insured or uninsured. Household income was coded into approximate quartiles.

Other explanatory variables comprised sex, age and tooth brushing. Age was coded into age groups of 30–39, 40–49 and 50–61 years. Tooth brushing was coded as those who brushed twice a day or more or those who brushed less than twice a day.

### Analysis

The analyses were restricted to dentate persons. Respondent characteristics were compared to population estimates derived from a nationally representative data collection (the 2010 National Dental Telephone Interview Survey) [31]. Unadjusted associations of SRDH were examined by the explanatory variables, followed by assessment of associations with dental insurance stratified by household income group. Adjusted associations between SRDH, insurance and household income were assessed in a multiple variable regression model. Prevalence ratios of good to excellent self-rated dental health adjusted for covariates were estimated using a log binomial model. The association between self-rated dental health and insurance stratified by household income group was assessed, followed by a model which included an interaction term for insurance and household income. These models assessed whether the relationship between SRDH and insurance was modified by income.

## Results

Responses were collected from n = 1,093 persons (response rate = 39.1%). Of these, 96% were dentate (n = 1,052).

More than half (57.7%) of the dentate respondents were female and just over 40% were in the 50–61 year age group. Just over one-half brushed their teeth twice a day or more. Distribution across the four household income groups was reasonably even and just over one-half had dental insurance (Table 1).

**Table 1:** Respondent characteristics compared to population estimates

	**Dentate respondents n = 1052**		^**(a)**^**Population estimates n = 4010**	
	**Per cent**	***(95% CIs)***	**Per cent**	***(95% CIs)***
**Sex**				
Male	42.3	*(39.3,45.3)*	49.8	*(47.6, 52.0)*
Female	57.7	*(54.7,60.7)*	50.2	*(48.0, 52.4)*
**Age group (years)**				
30 – 39	24.7	*(22.1,27.3)*	34.2	*(32.0, 36.6)*
40 – 49	32.9	*(30.0,35.7)*	32.7	*(30.7, 34.8)*
50 – 61	42.5	*(39.5,45.5)*	33.0	*(31.2, 35.0)*
**Tooth brushing**				
Less than twice a day	42.9	*(39.9,45.9)*	n.a.	
Twice a day or more	57.1	*(54.1,60.1)*	n.a.	
**Income group**				
<$40 000	20.7	*(18.2,23.3)*	21.7	*(19.3,24.2)*
$40 000 – < $80 000	31.6	*(28.7,34.5)*	33.0	*(30.4,35.7)*
$80 000+	47.7	*(44.5,50.7)*	45.3	*(42.5,48.2)*
$80 000 – < $120 000	26.0	*(23.2,28.7)*	n.a.	
$120 000+	21.7	*(19.1,24.2)*	n.a.	
**Dental insurance**				
Insured	53.9	*(43.1,49.1)*	59.1	*(56.9, 61.2)*
Uninsured	46.1	*(50.9,56.9)*	40.9	*(38.8, 43.1)*
**Self-rated dental health**				
Poor/fair	27.5	*(24.8,30.2)*	19.8	*(18.0, 21.8)*
Good to excellent	72.5	*(69.8,75.2)*	80.2	*(78.2, 82.0)*

^(a)^Population comparison estimates derived from the 2010 National Dental Telephone Interview Survey (NDTIS), participants aged 30–61 years. Income categories were not comparable with current study; in the 2010 NDTIS the highest income category was $110,000 or more.

n.a.: denotes not available.

Dentate respondents significantly varied from the population from which the sample was drawn. Respondents were more likely to be female, to be in the oldest age group (50–61 years), less likely to be insured for dental services and were more likely to report their SRDH as poor or fair (Table 1).

Nearly three-quarters (72.5%) of the dentate respondents rated their dental health as good, very good or excellent. SRDH did not vary by sex, but varied significantly by age group, tooth brushing frequency, household income and dental insurance status. Those in the oldest age group (50–61 years) had a lower proportion than younger age groups reporting good dental health. Those who usually brushed their teeth twice a day or more had a higher proportion with good dental health compared to those brushing less than twice a day. There was an observable gradient across the income groups with the highest household income group ($120,000+) reporting the highest proportion with good dental health. Insured adults had a higher proportion with good dental health compared to uninsured adults (Table 2).

**Table 2:** Self-rated dental health by respondent characteristics

	**Self-rated dental health: good to excellent**	
	**Per cent**	***(95% CIs)***
**Sex**		
Male	70.9	*(66.7,75.2)*
Female	73.6	*(70.1,77.1)*
**Age group (years)**		
30 – 39	75.9	*(70.6,81.1)*
40 – 49	76.8	*(72.3,81.3)*
50 – 61	67.0	*(62.6,71.4)*
**Tooth brushing**		
Less than twice a day	65.3	*(60.9,69.7)*
Twice a day or more	78.0	*(74.7,81.3)*
**Income group**		
<$40 000	53.9	*(47.1,60.7)*
$40 000 – < $80 000	73.8	*(68.9,78.7)*
$80 000 – < $120 000	78.2	*(73.2,83.3)*
$120 000+	82.8	*(77.7,87.8)*
**Dental insurance**		
Insured	79.9	*(76.5,83.2)*
Uninsured	63.8	*(59.6,68.1)*
**Total**	**72.5**	***(69.8,75.2)***

Dental insurance was also positively associated with household income, only a quarter (25.7%) of adults in the lowest income group (<$40,000) were insured for dental services compared to nearly three-quarters (72.6%) for adults in the highest income group ($120,000+).

**Figure 1:**
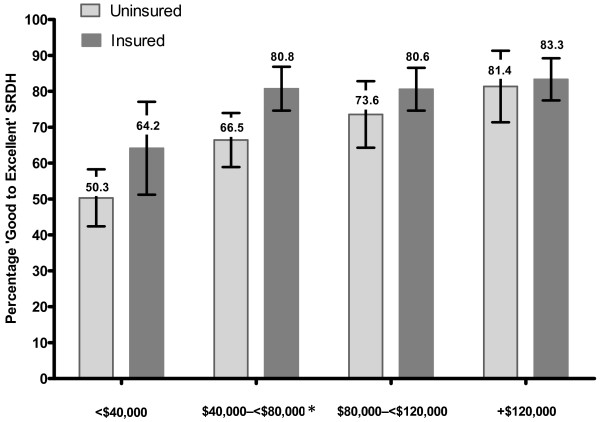
**Self-rated dental health by dental insurance status and household income group.** Note: * p < 0.05 Chi-square statistic.

Figure 1 illustrates the unadjusted associations between SRDH and insurance status. For respondents in the highest income group ($120,000+), the proportion rating their dental health as good to excellent did not vary by insurance status. In contrast, amongst adults in the lower income groups, the proportion reporting good dental health was higher for the insured; this difference was statistically significant for those in the $40,000– < $80,000 household income group (Figure 1).

Prevalence ratios (PR) of having good dental health were adjusted for dental insurance and other covariates (sex, age and tooth brushing) in a series of models stratified by household income group. Dental insurance was significantly associated with good dental health for the two lowest income groups but was not associated with good health for the two highest income groups (Table 3).

**Table 3:** Adjusted prevalence ratios of good to excellent self-rated dental health stratified by household income group

	**Income < $40,000**			**Income $40,000– < $80,000**			**Income $80,000– < $120,000**			**Income ≥ $120,000**		
	**PR**	***(95% CIs)***	**P**	**PR**	***(95% CIs)***	**P**	**PR**	***(95% CIs)***	**P**	**PR**	***(95% CIs)***	***P***
	n = 206			n = 314			n = 258			n = 215		
**Sex**												
Male	1.05	*(0.81,1.36)*	0.706	1.10	*(0.97,1.24)*	0.141	0.83	*(0.72,0.96)*	0.014	0.89	*(0.79,1.01)*	0.069
Female (ref.)	–			–			–			–		
**Age group**												
30 – 39 (ref.)	–			–			–			–		
40 – 49	0.89	*(0.65,1.20)*	0.439	1.03	*(0.89,1.19)*	0.678	1.10	*(0.94,1.28)*	0.239	0.95	*(0.81,1.12)*	0.555
50 – 61	0.70	*(0.51,0.95)*	0.023	0.93	*(0.8,1.09)*	0.372	1.05	*(0.90,1.23)*	0.525	0.81	*(0.68,0.97)*	0.024
**Tooth brushing**												
Less than twice a day (ref.)	–			–			–			–		
Twice a day or more	1.33	*(1.03,1.71)*	0.029	1.20	*(1.05,1.38)*	0.009	1.11	*(0.96,1.29)*	0.154	1.08	*(0.94,1.24)*	0.263
**Dental insurance**												
Insured	1.38	*(1.07,1.78)*	0.014	1.20	*(1.05,1.37)*	0.007	1.10	*(0.96,1.26)*	0.184	1.00	*(0.85,1.17)*	0.997
Uninsured (ref.)	–		.	–			–			–		

Notes:

PR: Prevalence Ratios.

Ref: reference category.

Prevalence ratios (PR) of having good dental health were adjusted for the main explanatory variables (dental insurance and income), other covariates (sex, age and tooth brushing) and the interaction term (between insurance and income). There was a significant interaction between income and insurance in their effects on SRDH; there was an observable gradient in the PR across income groups by insurance status. Insured adults in the two lowest income groups had a higher prevalence (33% and 21% respectively) of having good SRDH compared to the insured in the highest income group ($120,000+) (Table 4).

**Table 4:** Adjusted prevalence ratios of good to excellent self-rated dental health

	**PR**	***(95% CIs)***	**P**
**Sex**			
Male	0.91	*(0.85–0.99)*	0.024
Female (ref.)			.
**Age group**			
30 – 39 (ref.)	-		
40 – 49	1.00	*(0.92–1.09)*	0.968
50 – 61	0.89	*(0.82–0.98)*	0.012
**Tooth brushing**			
Less than twice a day (ref.)	-		
Twice a day or more	1.14	*(1.05–1.22)*	0.001
**Income group**			
<$40 000	0.59	*(0.49–0.72)*	<.000
$40 000– < $80 000	0.78	*(0.66–0.91)*	0.002
$80 000– < $120 000	0.84	*(0.71–1.00)*	0.051
$120 000+ (ref.)	-		.
**Dental insurance**			
Insured	0.96	*(0.85–1.11)*	0.627
Uninsured (ref.)	-		.
**Interaction**			
Insured × < $40 000	1.33	*(1.00–1.77)*	0.051
Insured × $40 000– < $80 000	1.21	*(1.01–1.46)*	0.043
Insured × $80 000– < $120 000	1.12	*(0.92–1.36)*	0.260

Notes:

PR: Prevalence Ratios.

Ref: reference category.

Omnibus test p < 0.000.

## Discussion

The results of this study showed that household income had a modifying effect on the association between dental insurance and self-rated dental health. Dental insurance was associated with self-rated dental health in the second lowest income household group ($40,000– < $80,000), but there was no association observed in the higher income groups.

The finding that household income was positively associated with self-rated dental health was consistent with previous studies [9,16-19]. The association between lower income and poorer self-rated oral health has been explained in three ways. Firstly, household income has a direct impact on the ability to access goods and services that promote dental health [16]. Individuals from lower income households are likely to lack sufficient economic resources to obtain timely and comprehensive dental care and may avoid or delay visiting a dentist until they experience dental problems or pain [11,15,19,22]. Secondly, individuals from lower socioeconomic backgrounds may be more likely to engage in risk behaviours that have a negative impact on their oral health, such as making poor dietary choices [16,17]. While a socioeconomic gradient has not been observed for dental self-care, challenging the commonly held view that personal neglect can explain the association between low income and poor oral health [14], self-care has been associated with SES gradients in disease through an interaction effect of SES, self-care and dental visiting on disease. An Australian study found that for those in lower income groups with no recent access to care, tooth brushing frequency was associated with untreated decay but there was no association between tooth brushing and disease for higher income groups or lower income groups with recent dental visits [32]. Finally, socioeconomic status has been linked to differences in psychosocial resources and psychological traits, which have an influence on health outcomes and the ability to cope with health problems [16,17]. It has been reported that self-esteem, life satisfaction, stress and depression partly explained socioeconomic disparities in self-rated oral health, suggesting that these factors may influence individuals’ response to and experience of oral health problems [16]. Adults with poor psychosocial scores were found to be more likely than their higher scoring counterparts to rate their oral health poorly, across all household income categories [9]. These factors could play an important role in understanding the association between income and self-rated dental health.

It has previously been reported that insured individuals were more likely than their uninsured counterparts to obtain regular and comprehensive dental care [31]. Uninsured individuals were more likely to have problem-oriented visiting patterns due to cost barriers and were less likely to receive preventive and regular care [11-14,21,24-26]. In this study dental insurance was found to be positively associated with self-rated dental health, which may be explained by the reduction in financial barriers to care that insurance provides. An American study found that higher out-of-pocket cost dental plans were associated with lower self-rated oral health [33]. Similarly, a Swedish study reported that patients enrolled in fee-for-service dental plans had worse oral health-related quality of life compared to those enrolled in an alternative system, which provided free dental care for a set annual fee [34]. It is possible that the greater reduction of financial barriers to dental care associated with some dental insurance plans may in part explain the associations with self-rated oral health.

However, it has often been suggested that selection bias may explain observed associations between insurance and access to care. For example, those with better self-rated dental health or a predisposition to seek dental care may be more likely to insure for dental services [35]. However studies controlling for potential confounders that may explain insurance effects on dental health and visiting, such as attitudes to dental care, or studies using analytical methods that minimise selection bias have found that insurance effects are only marginally attenuated and persist, therefore indicating that bias was likely to be minimal and not a large concern [26,35,36].

Household income modified the association between insurance status and self-rated dental health. Among lower income groups dental insurance was found to be positively associated with self-rated dental health, while there was no association in the highest income group. Previous studies have found that the positive impact of dental insurance on utilisation of dental services was most pronounced among lower income households [37,38]. Although adults from higher income households were found to be more likely to purchase dental insurance, the reduction in the reporting of financial barriers was greater among lower income groups [11,13,21,37].

### Study limitations

The cross-sectional nature of the analysis limits the ability to comment on the observed associations in terms of causal relationships. Furthermore, while the response yield provided sufficient numbers for analysis, the response rate was low, particularly with multiple follow-ups [39]. In addition, the respondents varied from the population from which the sample was drawn, limiting the generalisability of the estimates. The insured were underrepresented in the study sample, consequently, consistent with this underrepresentation, there was a higher percentage reporting poor to fair self-rated dental health in the sample compared to the comparison population. However, the main aim of this study was to explore associations between insurance, oral health and household income, not to generate population estimates. In addition, the associations explored were adjusted for the variables for which there were observed differences between the respondents and the population estimates. Lastly, there may be other unmeasured covariates associated with having both insurance and poor dental health that may mediate the observed associations (e.g. smoking status is associated with both having health insurance and self-rated dental health) [40].

The comprehensive and non-specific nature of self-rated health is considered an advantage in assessing dimensions of health in a different way to more guided questions, but it restricts the control over which aspects of health are emphasised [41]. However, self-rated health does comprise the underlying judgements of people that will likely guide their behaviours [42]. Furthermore, there is good correspondence between self-rated dental health and clinical measures such as caries and tooth loss, which support their validity [43].

## Conclusion

For lower socioeconomic groups being insured was associated with better self-rated dental health, but for adults in higher socioeconomic groups their self-rated dental health did not vary significantly by insurance status. Further studies with samples that are more representative of this population are required to assess the generalisability of this finding.

## Competing interests

The authors declare that they have no competing interests.

## Authors’ contributions

All authors were involved in the interpretation of data, revising the paper and giving final approval for publication of the manuscript. DNT collected and prepared data for analysis, conducted analysis, prepared results, and drafting of manuscript. OA contributed to the initial draft manuscript. DSB was involved in analysis and design of the project and drafting of the manuscript.
